# Successful cardiopulmonary resuscitation of cardiac arrest induced by massive pulmonary embolism under general anesthesia: a case report

**DOI:** 10.3389/fcvm.2023.1164076

**Published:** 2023-06-22

**Authors:** Zhen Li, Ning Cai

**Affiliations:** Department of Anesthesiology, Fuyang People's Hospital, Fuyang, China

**Keywords:** pulmonary embolism, general anesthesia, electrocardiography, computed tomography pulmonary angiography, thrombolysis

## Abstract

**Background:**

While pulmonary embolism (PE) is a common occurrence, a large life-threatening PE is not. Herein, we discuss the case of a patient with a life-threatening PE that occurred under general anesthesia.

**Case presentation:**

We present the case of a 59-year-old male patient who was at bed rest for several days due to trauma, which resulted in femoral and rib fractures and a lung contusion. The patient was scheduled for femoral fracture reduction and internal fixation under general anesthesia. After disinfection and surgical towel laying, there was a sudden occurrence of severe PE and cardiac arrest; the patient was successfully resuscitated. Computed tomography pulmonary angiography (CTPA) was performed to confirm the diagnosis, and the patient’s condition improved after thrombolytic therapy. Unfortunately, the patient’s family eventually discontinued treatment.

**Discussion:**

Massive PE frequently occurs suddenly, may endanger a patient’s life at any point in time, and cannot be diagnosed quickly on the basis of clinical manifestations. Although the vital signs fluctuate greatly and there is insufficient time to conduct more tests, some factors such as special disease history, electrocardiography, end-tidal carbon dioxide, and blood gas analysis may help us determine the preliminary diagnosis; however, the final diagnosis is made using CTPA. Current treatment options include thrombectomy, thrombolysis, and early anticoagulation, of which thrombolysis and early anticoagulation are the most feasible.

**Conclusion:**

Massive PE is a life-threatening disease that requires early diagnosis and timely treatment to save patients’ lives.

## Background

While several cases of pulmonary embolism (PE) have been reported in the literature, cases of large perioperative PE are rare. The incidence of PE is one case of PE occurring in every 1,000 people in the United States every year, and the incidence of perioperative PE is five times higher than that of non-perioperative PE (1). In most cases, the embolus of PE is small and may not even be detected or diagnosed in a timely manner owing to atypical symptoms or inadequate diagnostic conditions (2). Perioperative massive PE that occurs under general anesthesia is disastrous because it can present with varied symptoms in different patients and its diagnosis is difficult, especially in patients with hemodynamic fluctuations. If massive PE is not diagnosed quickly and patients do not receive timely treatment, death may be instant (3). Here, we report a case of sudden massive PE that occurred under general anesthesia.

## Case presentation

We report the case of a 59-year-old male patient who suffered a car accident, which caused pain in his left thigh, resulting in unconsciousness. After treatment at a local hospital, radiography revealed multiple fractures of the right ribs, mandible fracture, and brain contusions. The patient was transferred to the bone and joint trauma ward of our hospital for further treatment. A radiographic examination at our hospital revealed that the continuity of the left femoral cortex was interrupted, and there was displacement. After a partial examination, including tests for coagulation (prothrombin time,14s; activated partial thromboplastin time, 32.40 s; fibrinogen, 4.930 g/L) and D-dimer level (5.29 mg/L), the surgeon prepared the patient for internal fixation of the fractured femur. Because this patient and his family wanted the operation to be performed as soon as possible, no examination for deep venous thrombosis of the lower extremity was performed. In addition, the surgeon did not perform interventions such as anticoagulation.

The patient had no previous history of deep vein thrombosis, family history of sudden cardiac death, or cancer. He also had no comorbidities and was in a healthy state before the accident took place. Ever since the patient had been hospitalized, he had been bedridden. Owing to the pain caused by the fracture, the patient did not perform any lower limb movement in his bedridden condition.

**Figure 1:**
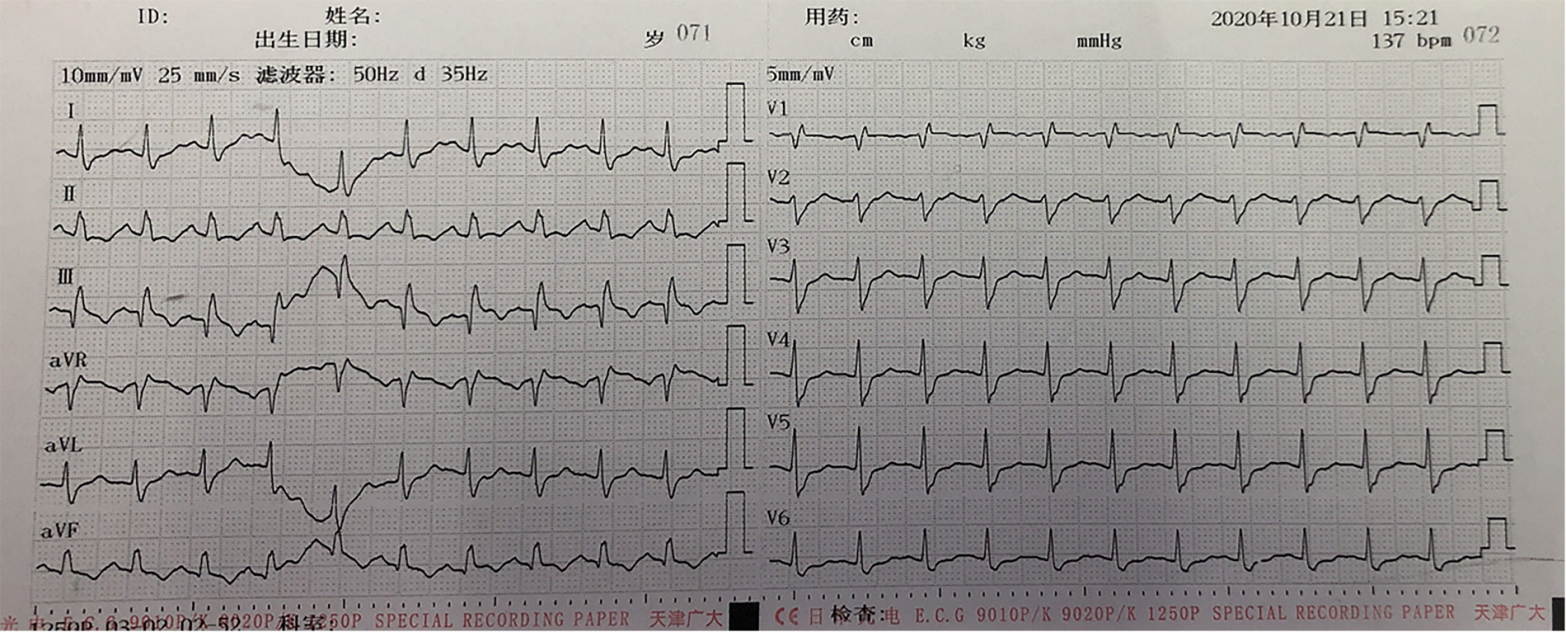
ECG of the patient after cardiopulmonary resuscitation: I-lead deep S wave (S1), III-lead Q wave (Q3), and T-wave negative (T3), which is called S1Q3T3.

**Figure 2.**
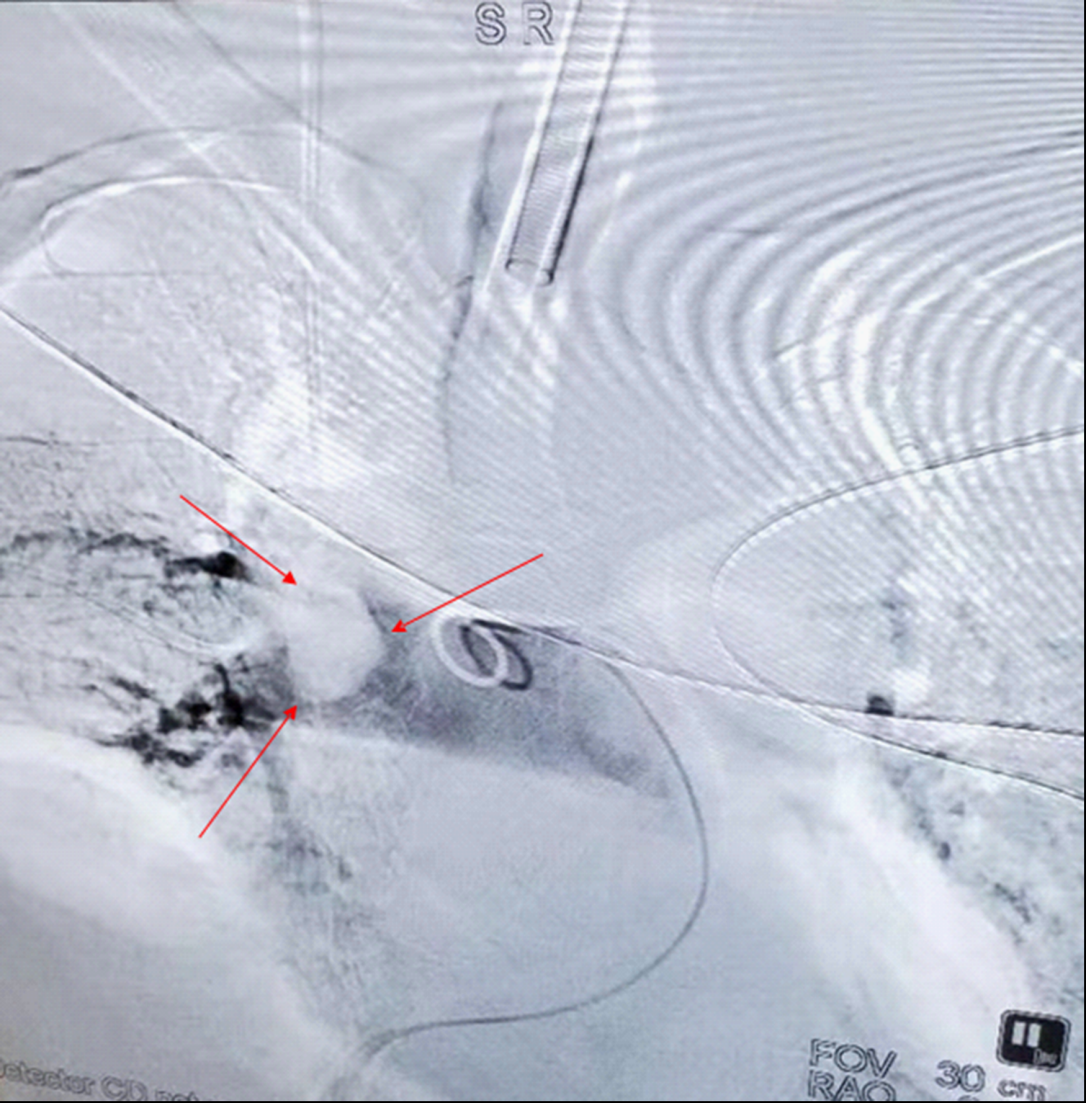
Computed tomography angiography of the lung approximately 3 h after hemodynamic fluctuations occurred: multiple embolisms in the two main pulmonary arteries and bilateral pulmonary artery branches. The arrows denote a very large embolus.

The patient’s anesthesia risk classification was American Society of Anesthesiologists grade III, cardiac function grade 2. In the operating room, arterial and internal jugular vein catheterizations were performed, and the arterial blood pressure (BP) was 140/80 mmHg. Sufentanil, etomidate, propofol, and cisatracurium were administered for routine anesthesia induction, which was performed smoothly, and tracheal intubation was successful. Propofol, remifentanil, and sevoflurane were used to maintain the depth of anesthesia, and the dosage was adjusted according to the vital signs. The surgeon then posed and sterilized the site and spread a sterile surgical towel. By the time the surgeon was ready to open the skin, the patient’s BP had suddenly dropped to 45/25 mmHg, approximately 1 h after the last induction time. When he was administered a short-order treatment with ephedrine and norepinephrine, his BP did not rise but tended to decrease. At this point, end-tidal carbon dioxide (P_ET_CO_2_) on the anesthesia monitor decreased significantly. An arterial blood gas analysis revealed a high partial pressure of carbon dioxide in the artery (PaCO_2_). BP continued to decrease, and the heart quickly stopped beating. Immediately after chest compressions, electrical defibrillation, and continuous high-dose epinephrine administration, the patient’s BP increased to approximately 105/65 mmHg, and the heart rate was 105 beats per minute. Cardiopulmonary resuscitation was performed twice, and BP was maintained at 110/65 mmHg and heart rate at 135 beats/min. During this period, the lowest end-tidal carbon dioxide was 12 mmHg, and the highest PaCO_2_ was 111 mmHg. Partial arterial oxygen pressure (PaO2) was 162 mmHg, the pH value was 6.99, and the lactate level was 11.7 mmol/L. Lead electrocardiography (ECG) revealed a complete right bundle branch block (RBBB), I-lead deep S wave (S1), III-lead Q wave (Q3), and T-wave negative (T3), which is called S1Q3T3. Figure 1 shows the ECG results. Computed tomography pulmonary angiography (CTPA) showed multiple embolisms in the two main pulmonary arteries and bilateral pulmonary artery branches (Figure 2). Blood gas analyses within 5 h after onset are presented in Table 1. Because of the urgency of the situation, an Echo-fast examination for quickly assessing the heart chambers was not performed after ECG and resuscitation.

**Table 1:** Blood gas analyses within 5 h after onset.

	13:02	14:06	14:27	14:44	14:56	15:15	15:44	16:08
PH (mmHg)	7.48	7.43	6.93	6.99	7.06	7.13	7.10	7.16
PCO_2_ (mmHg)	36	31	70	111	89	70	88	72
PO_2_ (mmHg)	85	266	262	162	213	192	186	196
Lac (mmol/L)	0.9	3.2	11.4	11.7	11.3	10.2	10.0	9.5
BEecf (mmol/L)	3.3	−3.7	−17.7	−4.7	−5.1	−5.9	−2.4	−3.0

The patient underwent inferior vena cava filter implantation and thrombolysis, for which the pulmonary artery catheter was used, followed by thrombolysis for several days in the intensive care unit. The patient’s vital signs gradually stabilized; follow-up on days 2, 3, and 4 showed that the patient responded correctly to their own name and simple instructions, and a gradual improvement in blood gas analysis results (Table 2). The relevant datasheet from the episode of care is given in Table 3. Unfortunately, the patient’s family sought discontinuation of treatment, following which the patient was voluntarily discharged.

**Table 2:** Arterial blood gas analysis.

	The first day 23:10	The next day 7:40
PH	7.279	7.439
PO_2_ (mmHg)	135	153
PCO_2_ (mmHg)	50.5	35.9
LAC (mmol/L)	6.5	3.0
PO_2_/FiO_2_	270	306

**Table 3:** Relevant datasheet from the episode of care.

Date	Body temperature (°C)	Heart rate (times per minute)	Respiratory rate (times per minute)	Blood pressure (mmHg)	Oxyhemoglobin saturation (%)	Urine volume (ml)
The first day	36.8	122	13	104/62	99	500
The next day	36.6	108	15	147/82	100	1,330
The third day	36.4	88	16	112/62	99	1,590
The fourth day	36.4	85	16	130/72	99	4,850

## Discussion

Massive PE is often sudden and harmful and may lead to a person’s death at any point in time. However, the initial symptoms may be atypical. In order to make the correct diagnosis and provide timely treatment, the exclusion criteria for PE need to be understood, and its possible clinical manifestations and related procedures, such as biochemical tests and imaging examinations, should be established.

Patients with suspected PE should be assessed for the clinical likelihood of PE using a validated risk score. In an article by Kruger et al, it was reported that a low or moderate clinical probability, combined with a normal D-dimer level, can rule out PE (4). A study by Salehi et al. provided evidence of suboptimal adherence to CTPA abandonment in patients who tested D-dimer-negative before CTPA was performed (5). Yang et al. observed that older age (increasing every 10 years), delay from the time of injury to surgery (daily), and fibrinogen levels  > 0. 4 g/L were independent risk factors for preoperative deep vein thrombosis (6), Therefore, it is worth considering early surgical treatment for such patients. When the dimer and fibrinogen levels increase, blood is in a hypercoagulable state. At this time, there is a high risk of thrombosis leading to serious consequences such as organ embolization; therefore, there needs to be vigilance when there is an increase in the dimer and fibrinogen levels.

Massive PE often needs to be distinguished from ST-segment elevation myocardial infarction as both conditions result in ST-segment elevation. The ECG of patients with PE can show an RBBB, leading to deep S wave (S1), III-lead Q wave (Q3), and T-wave negative (T3), called S1Q3T3, which indicates that the patient’s condition is serious and is the cause of the shock (7). In contrast, the ECG of patients with ST-segment elevation myocardial infarction does not show S1Q3T3.

When thromboembolic events are suspected during surgery, echocardiography will show right ventricular dilation and dysfunction with high specificity (8). Transesophageal echocardiography can be a reliable adjunct diagnostic measure, and PE should be considered when hemodynamic instability occurs and echocardiography shows right ventricular hypertension (9). Transthoracic echocardiography and transesophageal echocardiography are effective means to evaluate cardiac function status and provide a strong basis for the diagnosis and exclusion of some diseases, especially cardiopulmonary diseases.

CTPA is considered the gold standard technique in the emergency department for diagnosing patients with suspected acute PE (10). Early diagnosis of patients with suspected acute PE using emergency CTPA helps in reducing mortality in these patients (11). CTPA is mainly applicable to the examination of pulmonary vascular diseases, such as pulmonary embolism and pulmonary hypertension.

In our patients with endotracheal intubation under general anesthesia, airway pressure increased and P_ET_CO_2_ decreased, whereas arterial blood gas analysis revealed a significant increase in PaCO_2_; these results are consistent with those reported by Torres et al. (12).

The current PE management guidelines do not recommend specific treatments for patients with high-risk perioperative PE (13). However, in addition to anticoagulation, systemic and catheter thrombolysis are appropriate interventions for massive PE. Surgical pulmonary embolectomy can be performed to save patients’ lives, whereas right ventricular assist devices and extracorporeal life support can provide hemodynamic support in instances when removal of the embolus from a patient with PE is difficult (14).

Pulmonary embolism still needs to be differentiated from several diseases. Coronary heart disease is characterized by myocardial ischemia. Coronary angiography shows coronary atherosclerosis and obstruction of the lumen, but there is no pulmonary vascular obstruction. Most patients with aortic dissection have a history of hypertension, chest radiographs often show mediastinum widening, and chest computed tomography angiography shows aortic dissection.

Although a definitive diagnosis and effective treatment were the strengths of this study, significant omissions in thrombosis prevention and PE risk assessment during preoperative preparation, such as the absence of preoperative anticoagulation and lower-extremity deep vein thrombosis screening, acted as limitations. In patient cases such as ours, it is important to respect the feelings of the patient and his family, but the patient’s safety should be the paramount consideration.

## Conclusions

The diagnosis of perioperative massive PE is a major challenge for clinicians. In our patient, ECG showed an RBBB, S1Q3T3, decreased P_ET_CO_2_, and increased PaCO_2_. Transesophageal echocardiography showed hemodynamic instability and increased right ventricular resistance, all of which are helpful for the diagnosis of PE, which can be confirmed using CTPA when conditions permit, such as when the patient’s vital signs are stable or when the hospital has sufficient resources to perform CTPA. Early and rapid diagnosis, timely treatment, and saving patients’ lives are the treatment goals in patient cases such as ours.

## Data Availability

The original contributions presented in the study are included in the article; further inquiries can be directed to the corresponding author.
